# Synthesis, Characterization and Filtration Properties of Ecofriendly Fe_3_O_4_ Nanoparticles Derived from Olive Leaves Extract

**DOI:** 10.3390/ma14154306

**Published:** 2021-07-31

**Authors:** Djahida Boudouh, Rabia Ikram, Badrul Mohamed Jan, Hendrik Simon Cornelis Metselaar, Djamel Hamana, George Kenanakis

**Affiliations:** 1 Laboratoire de Technologie des Matériaux Avancés, Ecole Nationale Polytechnique de Constantine, BP 75A RP Ali Mendjeli, Constantine 25016, Algeria; djahidaboudouh@gmail.com (D.B.); d_hamana@yahoo.fr (D.H.); 2 Centre of Advanced Materials, Department of Mechanical Engineering, University of Malaya, Kuala Lumpur 50603, Malaysia; h.metselaar@um.edu.my; 3 Department of Chemical Engineering, University of Malaya, Kuala Lumpur 50603, Malaysia; 4 Laboratoire de Transformation de Phases, Université des Freres Mentouri—Constantine 1, Route de Ain El bey, Constantine 25017, Algeria; 5 Institute of Electronic Structure and Laser, Foundation for Research and Technology-Hellas, N. Plastira 100, Vasilika Vouton, GR-700 13 Heraklion, Greece; gkenanak@iesl.forth.gr

**Keywords:** olive leaves, Fe_3_O_4_ nanoparticles, characterization, WBDF, filtrate loss

## Abstract

Recently, value-added nanomaterials including nanoparticles or nanofluids have been significantly used in designing drilling fluids with tunable rheological properties to meet specific downhole and environmental requirements. In this work, we report novel water-based drilling fluids (WBDF) containing eco-friendly Fe_3_O_4_ nanoparticles (Fe_3_O_4_-NPs) prepared by using olive leaves extract (OLE) as a reducing and capping agent. A series of economical and excellent performance of WBDF was obtained by introducing low, medium, and high concentrations of Fe_3_O_4_-NPs into the conventional WBDF. The synthesis of Fe_3_O_4_-NPs was accomplished through the thermal decomposition of iron precursors in an organic medium. NPs were added to the based fluid at concentrations of 0.01, 0.1, and 0.5 wt%. Emission scanning microscopy (FESEM), field- and Fourier transform infrared spectroscopy (FTIR), X-ray diffraction (XRD), and Energy-dispersive X-ray analysis (EDX) were used for Fe_3_O_4_-NPs analysis. Compared to the conventional WBDF, the addition of Fe_3_O_4_-NPs as an additive in the based fluids has been investigated to help increasing viscosity and yield point, which is advantageous for hole cleaning, as well as decreasing fluid loss and mud cake thickness.

## 1. Introduction

Over the years, hydrocarbons have been traditionally the main source of substance to play a crucial role in the fulfillment of various industrial requirements [1]. With the projected increase in human population to 9.8 billion by 2030, an increase in the availability of goods and services is expected to be accomplished by increasing global gas and oil production [2]. Nanotechnology has been successfully used widely in many applications such as nanomedicine, nanoelectronics, and energy-related fields [3]. In addition, the usage of nanotechnology is up-surging in the oil and gas industry. Consequently, the industry has been able to successfully capitalise on improved drilling operations [4], enhanced oil recovery (EOR) [5], lubricity [6], corrosion inhibition, cementing, and reduction of heavy oil viscosity [7].

The stability, strength, and high thermal conductivity of nanomaterials is essential for the improvement of oil and gas downhole separations [8]. In this regard, nanotechnology contributes to the development and maintenance of drilling process equipment, allowing improved water and corrosion resistance, better reliability, stable rheological properties, and filtrate loss issues of wellbore instability [9,10]. The drilling industry has been revolutionized by the modifications of additives in drilling fluids to meet specific downhole and environmental requirements, as well as tunable rheological properties. Drilling fluids or drilling muds are classified into WBDF, oil-based drilling fluids (OBDF), and synthetic-based drilling fluids (SBDF) [11]. During the drilling process, mud circulation is one of the most critical systems of rotary drilling that is formulated to perform a wide range of functions, such as monitoring subsurface pressures, cleaning the wellbore, stabilizing exposed rock, providing buoyancy, cooling, and lubrication [12]. Such fluids must be constructed in a way that these can work effectively under intense downhole conditions with less formation damage. WBDF is the most common type of mud used in drilling operations globally. According to the literature, 80% of the world’s drilling wells have used WBDF [13]. Compared to OBDF and SBDF, WBDF are less expensive. Despite having higher operational efficiencies than WBDF, it has been observed that the use of OBDF and SBDF in drilling operations has decreased significantly due to environmental concerns [14]. On the other hand, WBDF have some drawbacks, including pipe sticking, poor lubricity, increased drag and torque, borehole erosion, gel formation, consolidation formation, wellbore instability, lost circulation, and shale swelling [15,16]. Hence, an improved formulation of WBDF is an alternative with efficient clay inhibition and enhanced rheological properties [17].

The selection of a suitable type of circulation fluid for drilling a well at a lower cost with minimal environmental risk and formation damage is among the most important criteria of the drilling process [18]. Drilling fluid is essentially a clay-water or oil mixture. Chemicals such as acids, polymers, and fibres are commonly added to drilling fluids to manipulate their functions and properties [19]. In general, the literature highlights the primary targets of these chemical additives, which are enhancing wellbore stability, improving wellbore cleaning, reducing fluid loss, and enhancing the rheological properties of the drilling fluids [11,19,20]. However, their application is limited due to environmental damage and high cost. Therefore, it is important to develop a new form of alternative chemicals and mud additives with low cost, high quality, are eco-friendly, and are readily biodegradable to avoid any short- or long-term environmental effects. Researchers have investigated the impact of nanofluid drilling on nearly all of the common and specific issues that based fluids are likely to encounter in the wellbore [20,21].

Numerous studies have claimed the addition of nanoparticles which improved drilling fluids by providing optimal rheological and filtration properties, increased shale stability, and enhanced wellbore strengthening as alternatives to costly chemicals [13,17,22]. The petroleum industry has been looking for polymers or natural products that are multifunctional, biodegradable, thermally stable, and environmentally friendly in order to build smart drilling fluids for petroleum exploration and extraction [23]. Due to their eco-friendly effects, plant extracts are preferred for nanoparticle synthesis. The preparation of NPs from various green materials has been used to achieve specific goals and is documented in the literature [24]. Thus, environmentally friendly and high-performance drilling fluids production is the focus of this research.

Metal oxide nanoparticles have been a strong candidate to be used as an additive in drilling fluids, due to their high surface area to volume ratio and remarkable heat transfer, plugging, and coating properties [25]. Researchers have investigated the transport, attachment, and retention of SiO_2_ NPs in core plugs at different base fluid salinity (0–3 wt% NaCl). The hydrophilic SiO_2_ NPs were injected into the limestone core as nanofluid of various sizes (5 nm and 20 nm) and concentrations (0.005–0.1 wt%) at various temperatures (23 and 50 °C) [26]. It was found that SiO_2_ NPs dispersed in brine solution of (NaCl) to be gradually retained in the limestone core as the ionic strength of the solution increased. Further research has been conducted to investigate the effects of CuO and ZnO with sizes lower than 50 nm as an additive in WBDF with xanthan gum aqueous solution as the base fluid [27]. When compared to WBDF, nanoparticle-based drilling fluids have improved electrical and thermal properties by approximately 35%, while other studies highlighted the enhancement of rheological and filtration properties, as well as thermal conductivity of bentonite-based drilling fluids using copper oxide/polyacrylamide nanocomposite which used standard methods for both salty and deionized water [28]. The idea of using Ferro Fluid called “smart-nano fluid,” that contains surfactants as enhanced flooding in EOR processes resulted with their findings particularly on Ferro Fluid’s rheological properties [29]. Following that, the effect of CM Fe_3_O_4_-NPs on the properties of aqueous bentonite suspension at HP/HT conditions was reported [30]. Researchers found that the addition of CM Fe_3_O_4_ NPs increased the ability to control filtration loss with a thin and impermeable mud filter cake. Various types of NP flooding have been investigated, and the results show that Fe_2_O_3_ NPs have a higher recovery factor in distilled water than in oil [31].

Owing to that, research studies have discussed the effect of iron oxide NPs on the rheological and filtration properties of WBDF. However, none of the above-mentioned studies tried to perform a comparative evaluation when adding Fe_3_O_4_-NPs prepared using plant extracts.

It is noteworthy that olive leaves contain a variety of potentially bioactive chemicals, including hydroxytyrosol and oleuropein fragments, which may have antioxidant properties [22,31]. Based on earlier studies, it is not clear how the concentration of Fe_3_O_4_-NPs additives could affect the overall performance of the rheological and filtration properties. This study describes the green synthesis of Fe3O4 nanoparticles using OLE, which contains a variety of polyphenols that can act as reducing and capping agents. Therefore, this work attempts to fulfill this research gap by proposing environmentally friendly olive-leaves-derived Fe_3_O_4_-NPs and studying the effect of drilling fluids, water bentonite suspensions, in terms of rheological and fluid loss properties when incorporating Fe_3_O_4_-NPs prepared at three different levels (0.01, 0.1, and 0.5 wt%). Firstly, Fe_3_O_4_ nanoparticles were prepared by a facile and eco-friendly method using olive leaves extraction (OLE). Secondly, X-ray diffraction (XRD), field-emission scanning electron microscopy (FESEM), and Fourier transform infrared spectroscopy (FTIR) were used for the characterization of synthesized NPs, and finally, Fe_3_O_4_-NPs were tested for their ability to provide rheological control by exploring their rheological properties.

## 2. Experimental Section

### 2.1. Materials and Methods

Iron (III) nitrate hydrate (Fe(NO_3_)_3_.9H_2_O, 99% from Spectrum). Ethanol (99.8%) from ChemPur. Olive leaves are collected from the high Aures mountains- Batna province, Algeria.

Green drilling fluid components were used: NaOH (purity ≥ 99 wt%), KCl (purity ≥ 99 wt%), and Carboxymethylcellulose (purity ≥ 99 wt%), were acquired from Sigma-Aldrich, St. Louis, MO, USA. Bentonite and barite (purity 91–93 wt%) were provided by Merck, Germany. Distilled water was used to prepare all aqueous solutions with no further purification.

#### 2.1.1. Olive Leaves Extract Preparation

**Figure 1:**
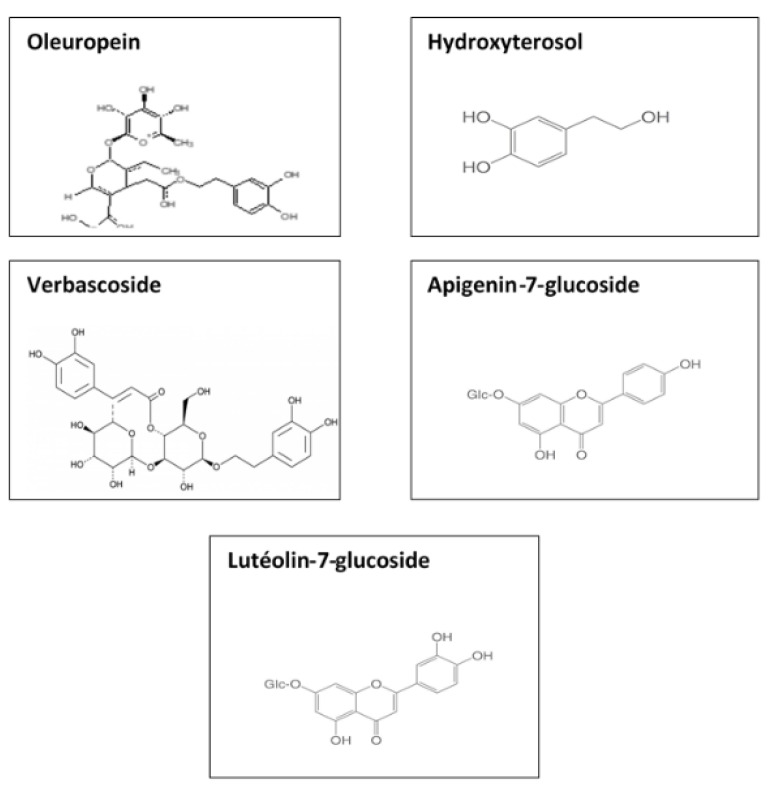
Common reducing agents of olive leaves extract.

Olive leaves were collected in the autumn season, washed with distilled water to remove impurities, cut into small pieces, and dried for 7 days. An amount of 10 g of the leaves were boiled in 100 mL of distilled water until the color of the aqueous solution turned to a green color. After that, the extract was cooled in air, filtered, and stored in a tight container at 4 °C for further utilization. The phenols present in olive leaves are mainly Hydroxytyrosol, Tyrosol, Catechin, Caffeic Acid, Vanillic Acid, Vanillin, Rutin, Luteolin-7-glucoside, Verbascoside, Apigenin-7-glucoside, Diosmetin-7-glucoside, Oleuropein, and Luteolein, as presented in Figure 1.

#### 2.1.2. Synthesis of Fe_3_O_4_-NPs

**Figure 2:**
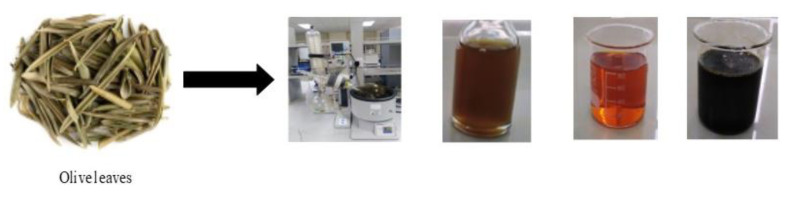
Synthesis of olive leaves-derived nanoparticles.

OLE was added to a solution of 0.6M Fe(NO_3_)_3_ in a (1:1) volume ratio to obtain a black colloidal solution for Fe_3_O_4_-NPs formation using OLE. At temperature of 50 °C, the mixture was then reflexed for 2 h. After 1 h of calcination at 550 °C in a tube furnace with pure argon gas, the magnetic black powder was obtained as presented in Figure 2.

#### 2.1.3. WBDF Preparation Using Different Concentrations of Fe_3_O_4_-NPs

Based fluid was prepared in accordance with API recommended practice 13B-2 standards.

Table 1 shows the formulation used for the preparation of based fluid. Firstly, a bentonite slurry was made by mixing 25 g of bentonite powder with water for 20 min in a Hamilton Beach mixer. The slurry was then kept for 24 h to allow the bentonite to completely hydrate [32].

The pre-hydrated bentonite slurry was added gradually into water under mechanical stirring for 15 min. Furthermore, sodium hydroxide (NaOH) as a PH adjuster [33], Carboxymethylcellulose (CMC) as a viscofier for fluid loss control, potassium chloride (KCl), and barite as a weighing agent to help in holding the cuttings [34] were added into bentonite-based mud and kept stirred to homogenize the mixture.

**Figure 3:**
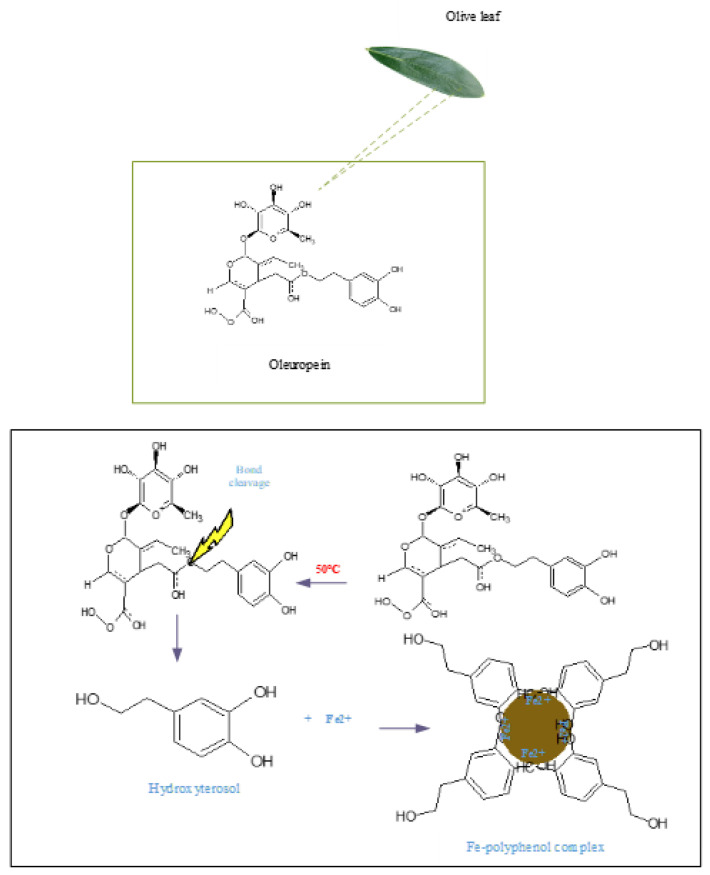
Schematic illustration of synthesis of Fe_3_O_4_ nanoparticles from the interaction between iron nitrate hydrate and OLE (reproduced with permission from Elsevier [22]).

Fe_3_O_4_-NPs concentrations of 0.01, 0.1, and 0.5 g were dispersed in the prepared aqueous solution and vigorously mixed for 15 min with the help of a mechanical stirrer to achieve uniform particle distribution for the production of nano-fluids (Table 1). Figure 3 represents the reaction mechanism for the synthesis of Fe_3_O_4_ from the interaction between iron nitrate hydrate and OLE.

### 2.2. Characterization

#### 2.2.1. Fe_3_O_4_-NPs Analysis

X-ray diffraction was carried out to investigate the crystallinity, phase composition, and purity as well as the average size of the Fe_3_O_4_-NPs, synthesized using a PANalytical X’Pert PRO X-ray diffractometer (XRD), (λ = 1.54 Å Cu Kα at 40 kV and 20 mA) in the 2θ range from 10° to 90°. A uniform layer of Fe_3_O_4_ powder was added to the holder to ensure that the surface was flat and smooth. The sample was analyzed for around 15 min. In a high vacuum mode, the morphology and particle dispersion were observed using a field-emission scanning electron microscope (FESEM) (AURIGA, made by ZEISS, UK) equipped with an energy-dispersive X-ray spectroscope (EDX). The investigation of the potential bonding between Fe_3_O_4_NPs and OLE was conducted by using FTIR spectroscopy. This was to investigate the underlying factors that may influence the improvement of drilling fluid efficiency during drilling operations with the addition of Fe_3_O_4_ NPs. The structure of Fe_3_O_4_ prepared using OLE was studied using Fourier transform infrared (FTIR), with spectra obtained in the 400–4000 cm^−1^ range using a Nicolet iS10 FT-IR Spectrometer, UK.

#### 2.2.2. Rheological Properties Investigation

Fann Model 35 Viscometer (Houston, TX, USA) and Anton Paar rheometer (Germany) were operated at room temperature to measure the yield point (YP), plastic viscosity (PV), apparent viscosity (AV), 10 s, and 10 min gel strengths (GS) of WBDF. The mud sample was poured into the testing cup, and the rotor sleeve was immersed to the scribe line precisely with the sleeve rotating at 600 rpm and 300 rpm, waiting for dial readings to reach steady values which recorded the dial readings (Φ600) and (Φ300), respectively.

Relations below are used to obtain the desired parameters:

Plastic Viscosity, μ_p_ (cP): μp = Φ_6__00_ − Φ_3__00_

Apparent Viscosity, μ_a_ (cP): μa = (Φ_6__00_)/2

Yield Point, Y_b_ (lb/100ft^2^): Yb = Φ_3__00_ − μ_p_

Gel strength is the values of the maximum dial reading attained at 3 rpm after keeping the fluid undisturbed for a specific time, 10 s and 10 min for 10 s gel strength and 10 min gel strength, respectively.

#### 2.2.3. Filtration Properties

A filtration test was conducted by pouring the mud sample into the cell to within 1/2 inch of the top, and the filtrate was collected using a dry graduated cylinder placed under the drain tube. OFITE filter press was used for this test. The system used N_2_ to supply pressure and a standard filter paper. The pressure relief valve was opened and began to record filtrate volume in the function of time. According to the API recommendation for this test, the operating pressure was 100 psi and the temperature was atmospheric (77 °F). After 30 min, the filtrate volume was measured in cubic centimetres (to 0.1 ccs). The filter cake thickness was measured using digital Vernier caliper model Mitutoyo 500-197-20, to the nearest 1/32 inch.

## 3. Results and Discussion

### 3.1. XRD Analysis

Powder X-ray diffraction was conducted to investigate the crystalline nature, phase purity, as well as average size of the Fe_3_O_4_-NPs, synthesized using OLE.

X-ray diffraction was carried out to investigate the phase purity, crystalline nature, as well as the average size of the Fe_3_O_4_-NPs, synthesized using a PANalytical X’Pert PRO (Almelo, the Netherlands) X-ray diffractometer (XRD), (λ = 1.54 Å Cu Kα at 40 kV and 20 mA) in the 2θ range from 10° to 90°.

**Figure 4:**
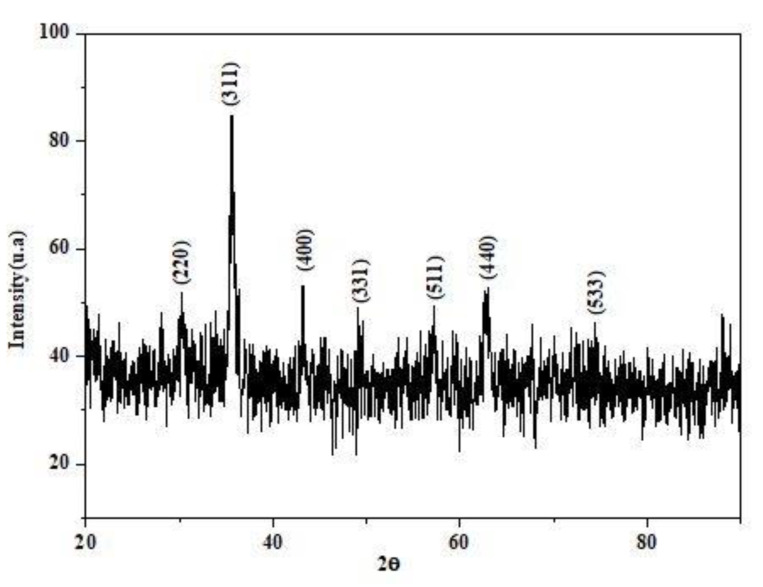
XRD Spectrum of Fe_3_O_4_-NPs.

Figure 4 shows the XRD pattern of synthesized nanoparticles. The diffraction peaks appeared at 2θ = 30.16°, 35.52°, 43.17°, 47.27°, 57.1°, 62.7°, and 74.2°, corresponding to the crystal planes (220), (311), (400), (331), (511), (440), and (533), respectively. The analyzed diffraction peaks that have a cubic phase matched well with the standard magnetite XRD patterns (JCPDS file No.96-900-5839).

There were no peaks of other iron compounds discovered, indicating that Fe_3_O_4_ is extremely stable. Furthermore, it verifies that no other components were formed during the reflexing or heating processes. Thus, the appearance of sharp pics confirms the complete formation of high crystallinity Fe_3_O_4_-NPs.

The formula of Debye–Scherrer $D=\frac{K*\lambda }{\beta *\cos\theta }$ was used to calculate the average crystal size of Fe_3_O_4_-NPs [35].

Where D is the size of synthesized Fe_3_O_4_-NPs crystallite, K is Scherrer constant of 0.9, λ is the X-ray radiation wavelength of Cu Kα (0.154 nm), β_hkl_ is the full-width at half maximum (FWHM) expressed in radian, and θ_hkl_ is the diffraction angle. Full-width-at-half-maximum (FWHM) of the intense peak (3 1 1) was used and the crystallite size was found to be 15.3 nm.

### 3.2. FESEM Analysis

The examination of the morphology of the prepared Fe_3_O_4_-NPs was conducted by using field-emission scanning electron microscopy (FESEM) with energy-dispersive X-ray spectroscopy (EDX).

**Figure 5:**
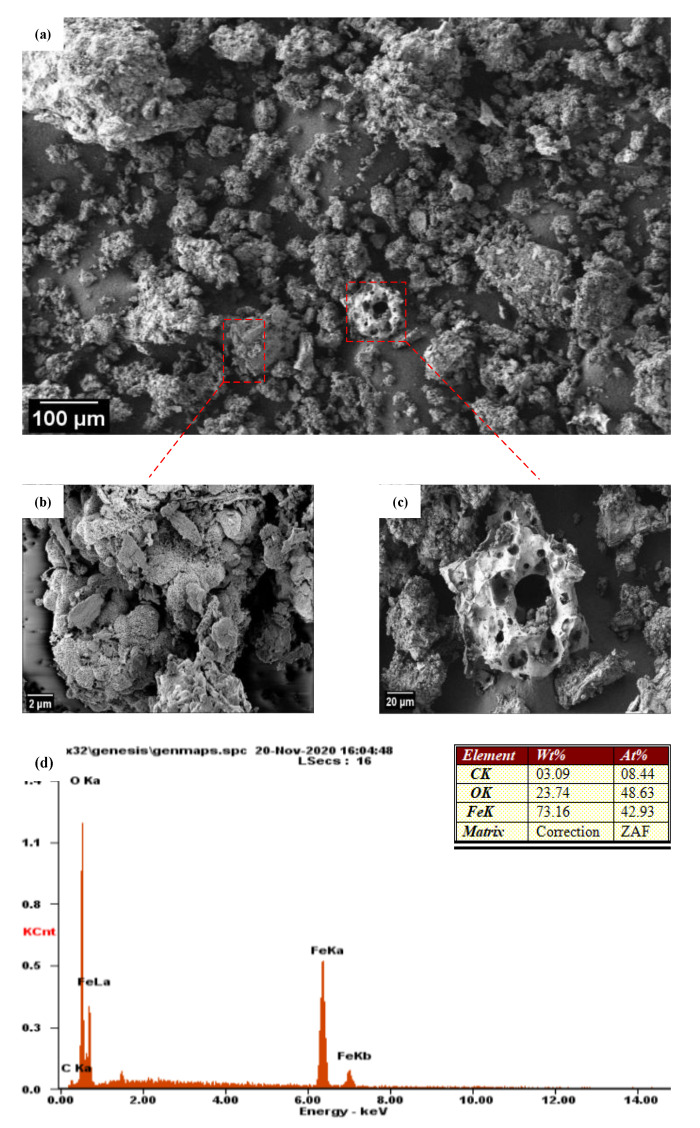
FESEM imaging (**a**–**c**), and EDX (**d**) of the Fe_3_O_4_-NPs.

Figure 5a shows the rough and hard surface of Fe_3_O_4_-NPs comprising numerous heterogeneous agglomerates in flake shape and cavity-like structures as highlighted in Figure 5b,c, respectively.

Figure 5b displays that Fe_3_O_4_-NPs had a strong tendency to aggregate, resulting in a cluster formation, which was because of the Fe_3_O_4_’s small size (15.3 nm), according to XRD results.

Figure 5d represents the EDX of Fe_3_O_4_-NPs, which indicates the presence of Fe and O in an atomic ratio of approximately 3:4 in the NPs system besides the small amount of carbon which may be due to the carbonization of organic matter present in OLE after calcination process at 550 °C.

### 3.3. FTIR Study

**Figure 6:**
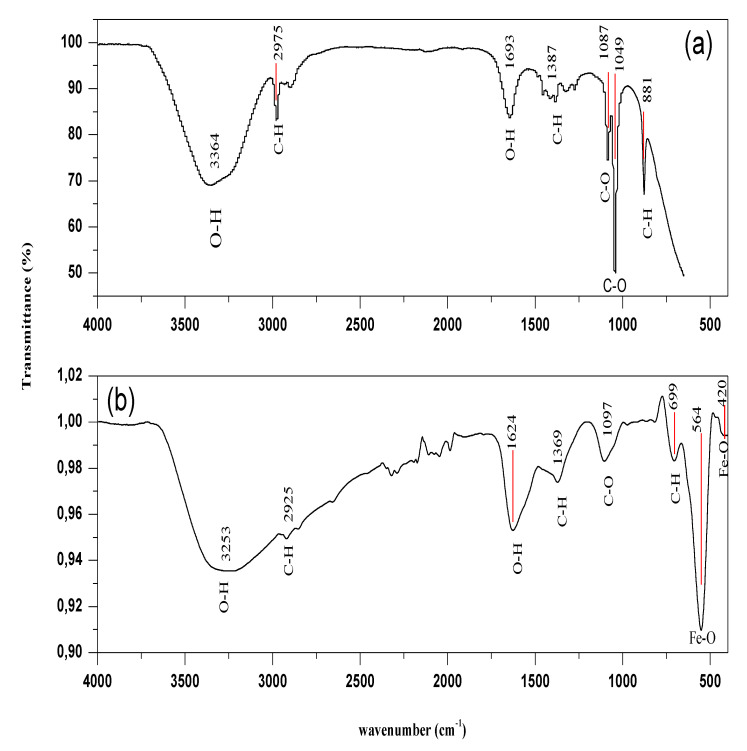
FT-IR analyses of OLE (**a**) and Fe_3_O_4_ nanoparticles (**b**).

The Fourier transform infrared (FTIR) spectroscopy analysis of OLE and Fe_3_O_4_-NPs is presented in Figure 6. The investigation of the nature of the chemical bonding between Fe_3_O_4_ particles and OLE was carried out by FTIR spectroscopy, with spectra obtained within the range of 400–4000 cm^−1^.

In Figure 6a, the spectra of OLE showed strong absorption bands at 3364, 2975, 1693, 1387, 1087,1049, and 881 cm^−1^, while absorption bands of synthesized Fe_3_O_4_-NPs were present at 3253, 2925, 1627, 1369, 1097, 699, 564, and 420 cm^−1^ (Figure 6b).

The O-H stretching vibration modes of free or adsorbed water and OH groups on magnetite particles’ surfaces are responsible for the broadband observed at 3364 cm^−1^ in the OLE. The IR band at 1387 cm^−1^ could very well be a phenolic OH bending.

According to Figure 6a, the absorption band at around 1693 cm^−1^ is associated with carbonyl stretching C = O (usually, peaks around 1700 indicate C = O, either from the sample or adsorbed CO_2_). C-H stretching and bending had lower peak intensities at 2975 and 881 cm^−1^, respectively. The -CH_2_ functional group is indicated by these C-H bands [36]. Aromatic compounds are commonly found in plants. The presence of a C-O stretching vibration band is indicated by the absorption peaks at around 1087 and 1049 cm^−1^ (of secondary and primary alcohol groups, respectively). From the FT-IR spectra of the synthesized nanoparticles using OLE, Figure 6b clearly narrates that the peaks located at 3253, 2925, 1624, 1369, 1097, and 699 cm^−1^ were a little bit shifted, confirming the interaction between OLE and nanoparticles. The spectra of synthesised Fe_3_O_4_-NPs, on the other hand, show two distinct sharp peaks at 564 and 420 cm^−1^. Fe_3_O_4_ is responsible for absorption peaks that appear in the 400–600 cm^−1^ range [37]. Hence, the Fe-O stretching vibration band is assigned to both absorption bands. Hence, the FT-IR results indicated the bonding types between OLE and Fe_3_O_4_-NPs.

### 3.4. Effect of Fe_3_O_4_-NPs Concentration on Drilling Fluid: Rheological Properties

**Figure 7:**
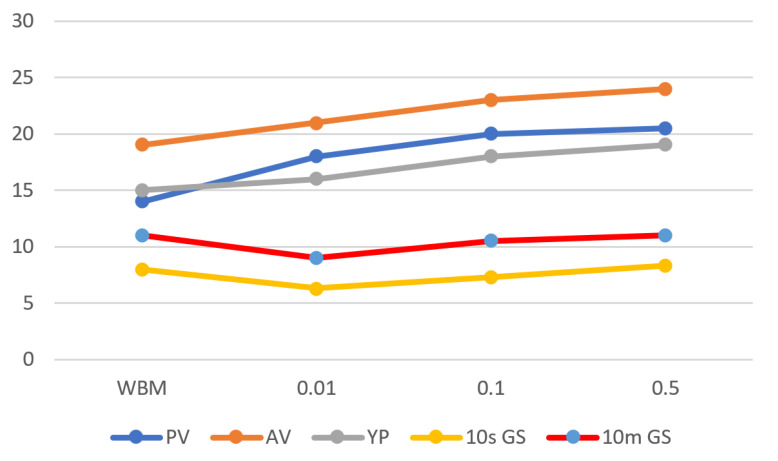
Rheological properties variation of WBM in function with Fe_3_O_4_-NPs addition.

Drilling fluid rheology is a critical function that influences many aspects of the drilling process. The most important rheological properties are likely apparent viscosity, plastic viscosity, yield point, and fluid gel strength, which were well achieved in this work by incorporating Fe_3_O_4_-NPs with WBDF, as shown in Table 2 and Figure 7.

#### 3.4.1. Plastic Viscosity

The optimum value of PV should be achieved by taking into account all operating conditions and the necessary mud characteristics for safe drilling operations.

**Figure 8:**
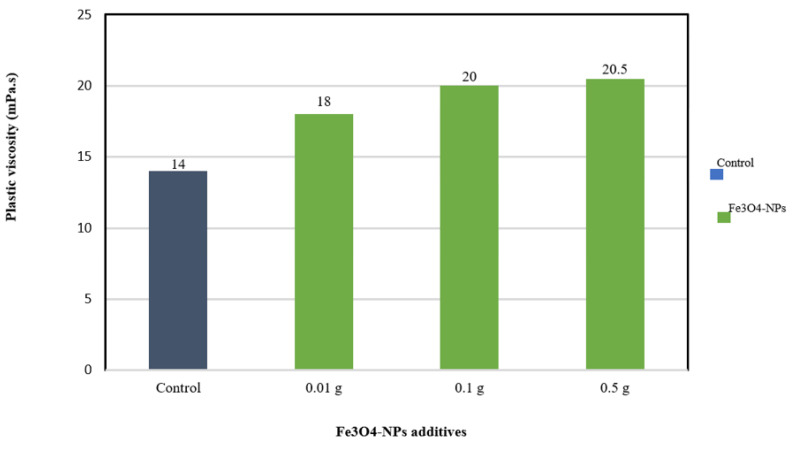
Plastic viscosity improvement of WBDF with the addition of Fe_3_O_4_-NPs.

Figure 8 shows the variation of viscosity of the WBDF and nanofluids (NF) at 25 °C upon addition of 0.01, 0.1, and 0.5 wt% Fe_3_O_4_-NPs.

The addition of 0.5 wt% Fe_3_O_4_-NPs showed a greater impact on the bentonite solution’s viscosity than the 0.01 and 0.1 wt% Fe_3_O_4_-NPs had, compared to the bentonite control. The viscosity-increasing behavior with the addition of Fe_3_O_4_-NPs can be related to multiple mechanisms that are mostly dependent on the characteristics of nanoparticles and their optimal dispersion within the platelets of clay forming a continuous phase of the mud system [38]. At the same dispersed particle volume concentration, it is well known that the nanofluid’s viscosity is much higher than the traditional dispersions viscosity. Once NPs are incorporated in the fluid, friction between layers of fluid may increase, causing the nanofluid viscosity to increase [39]. Furthermore, NPs act as a bridge between the clay platelets, due to their high surface area to volume ratio, and positively charged surface, resulting in strong attractive forces between the positive surface of Fe_3_O_4_-NPs and the clay particles negative charged area [40]. The viscosity of each fluid is ascribed to the mode of interaction between either the clay platelets and the surface charges of NPs or between the intercalated clay platelets themselves. Fe_3_O_4_-NPs promoted positive charges on the surface, which was induced by the wrapping of polyphenolic compounds present in the OLE around the nanoparticles during the preparation process (as illustrated in the graphical abstract). Thus, the addition of Fe_3_O_4_-NPs to the WBDF encouraged favorable charges on the negative bentonite platelet charges [41]. This attraction between Fe_3_O_4_-NPs and the bentonite clay platelet could be classified into three different categories: the attraction between the edges (E-E) as well as the attraction between faces (F-F) and attraction between edge and face (E-F), resulting in the creation of a network between Fe_3_O_4_-NPs and the bentonite clay platelets; this phenomenon is known as heterocoagulation [42].

FTIR spectroscopy was used to verify whether there were any possible bonding effects between Fe_3_O_4_-NPs and OLE, while the dispersion and the special heterogeneous morphological structures of nanoparticles were investigated by using field emission scanning electron microscopy (FESEM). FTIR and FESEM findings confirm the hypothesis of the viscosity increase.

#### 3.4.2. Yield Point

**Figure 9:**
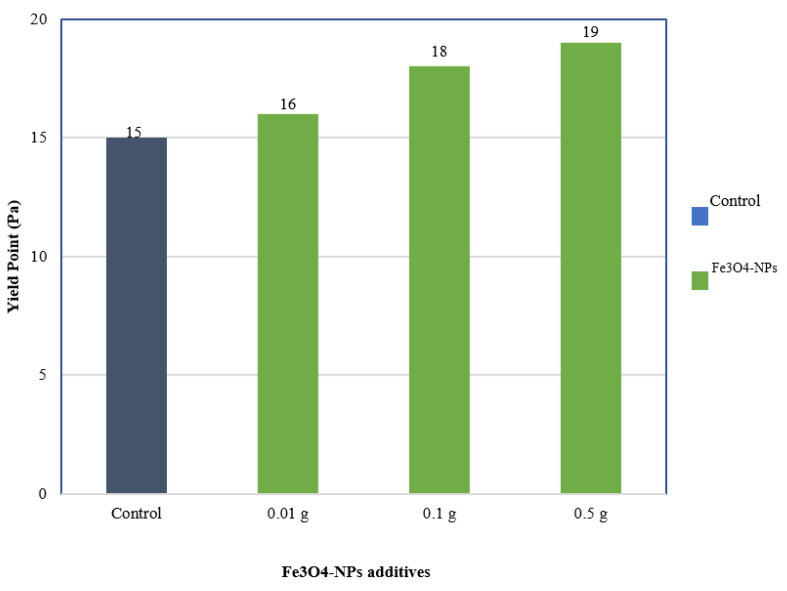
Yield point improvement of WBDF with the addition of Fe_3_O_4_-NPs.

Yield Point (YP) is defined as fluid flow resistance due to electrochemical forces within the fluid. The electrical charges were caused by the electrochemical forces on the reactive particles surface [38]. The mud’s ability to remove cuttings from the annulus under difficult conditions was evaluated by its yield point. The effects of adding 0.01, 0.1, and 0.5 wt% NPs on the yield point of WBDF are depicted in Figure 9.

Increased NPs concentrations result in better WBFs yield point performance. The maximum value of yield point was achieved 19 Pa at a concentration of 0.5 wt% compared to the bentonite control. The yield point-increasing behavior with the addition of Fe_3_O_4_-NPs can be related to the network structure formation between nanoparticles and bentonite clay.

This network improved fluid viscosity and flow resistance, as evidenced by a nearly one-order-of-magnitude increase in yield stress compared to the bentonite control, as shown in Figure 9. These findings are consistent with previous outcomes [43].

#### 3.4.3. Gel Strength

Gel strength tests the drilling fluid’s ability to keep the drill cuttings in suspension when circulation is stopped [38].

**Figure 10:**
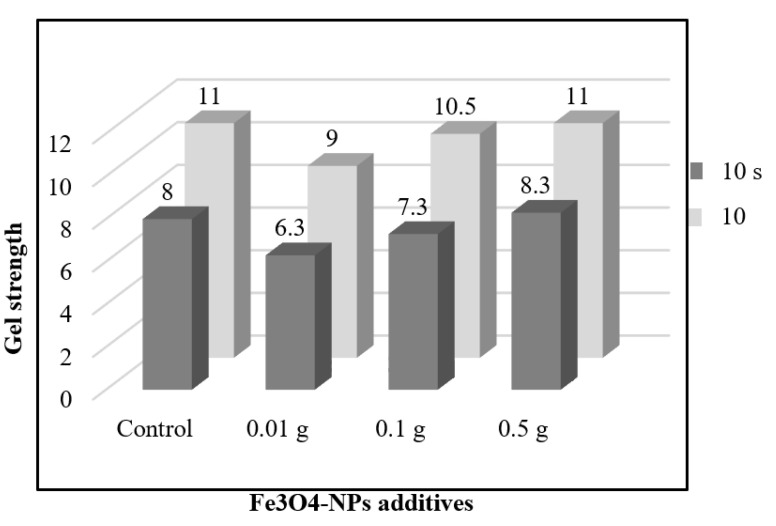
Gel strength of the prepared Fe_3_O_4_-NPs drilling fluids.

As shown in Figure 10, 0.5 wt% Fe_3_O_4_-NPs WBM have displayed sufficient gel strength at both 10 s and 10 min. The 10 s gel strength of WBDF was found to be constant (11 Pa) even after the addition of 0.5 g of Fe_3_O_4_-NPs, while the 10 min gel strength of WBDF increased slightly from 8 Pa to 8.3 Pa with 0.5 g Fe_3_O_4_-NPs. Hence, the function as a chain linking point between bentonite platelets that facilitates the gelation behavior of Fe_3_O_4_-NPs drilling fluid may be the reason behind that improvement (as explained in Section 3.4.1). On the other side, 10 s gel strength of WBDF was reduced from 8 Pa to 7.3, and 6.3 Pa with the addition of 0.1, and 0.01 g of Fe_3_O_4_-NPs, respectively. The same observation could be obtained for 10 min gel strength of WBDF, which was decreased with the incorporation of medium and low concentration of Fe_3_O_4_-NPs. This may be explained by the insufficient concentrations of 0.1 and 0.01 g of Fe_3_O_4_-NPs for the formation of gelation behavior between bentonite platelets.

### 3.5. Effect of Fe_3_O_4_-NPs Concentration on Drilling Fluid: Fluid Filtration Loss

**Figure 11:**
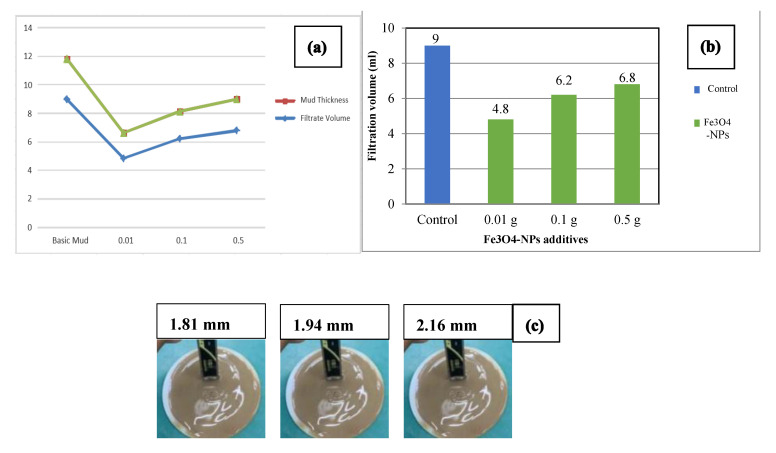
(**a–c**) Fluid loss and mud thickness using different Fe_3_O_4_-NPs concentrations.

Figure 11a presents the impact of Fe_3_O_4_-NPs drilling fluids on the filtration performance and the filter cake thickness at different concentrations.

The WBDF has shown a filtrate loss volume of 9 mL. The fluid loss has been significantly decreased into 6.8, 6.2, and 4.8 mL after the addition of 0.5, 0.1, and 0.01 wt% of Fe_3_O_4_-NPs to the WBDF, respectively, as shown in Figure 11b. Furthermore, as shown in Figure 11c, a decrease in fluid loss volume results in a decrease in filter cake thickness. It is clear that by adding the lowest Fe_3_O_4_-NPs concentration (0.01 wt%) to the mud, the smallest fluid loss volume was established and the thinnest mud cake was obtained, demonstrating great enhancement by lowering the filtrate volume by 46.6% when compared to WBDF, and hence this concentration could be optimal, and at this concentration, Fe_3_O_4_-NPs contribute to filling the nano- and micro-gaps in the filter cake [44,45]. As a result, Fe_3_O_4_-NPs were critical in blocking the nano-pores in the filter cake made of bentonite particles.

## 4. Conclusions

In this work, novel drilling fluids containing Fe_3_O_4_-NPs were prepared via green synthesis using OLE. Three different concentrations, low (0.01 wt%), medium (0.5 wt%), and large (0.5 wt%) of Fe_3_O_4_-NPs, were tested and evaluated for their capacity to give in situ rheological controllability at ordinary conditions. The following conclusions can be drawn based on the results obtained:

➢ The effect of Fe_3_O_4_-NPs concentration on the rheological property and fluid filtration was investigated.
➢ The best performance in both mud cake thickness and filtrate loss enhancement was obtained with the lowest concentration of Fe_3_O_4_-NPs.
➢ In an aqueous setting, the Fe_3_O_4_-NPs exhibit positive charges due to the phenolic compounds coated the nanoparticles, which would attract the bentonite clay platelets’ negatively charged surfaces.
➢ Coagulation was induced by the addition of Fe_3_O_4_-NPs to the WBM; the collective behavior of various types of clay particles were induced by the addition of an electrolyte to the clay solution, promoting a linked structure.
➢ The linked structure allows more water to be trapped between the layers. This causes an increase in viscosity and yield stress while reducing fluid filtration.
➢ The addition of Fe_3_O_4_-NPs reduced fluid loss volume, resulting in a thinner filter cake.

## Figures and Tables

**Table 1:** Preparation of based fluid.

Components	Amount (Concentration)
Distilled water (mL)	259.65
Pre-hydrated bentonite slurry (g)	25.0
NaOH (g)	0.15
CMC (g)	1.60
KCl (g)	25.0
Barite (g)	65.41
Fe_3_O_4_-NPs (g)	0.01, 0.1, 0.5

NaOH: Sodium hydroxide. CMC: Carboxymethylcellulose. KCL: Potassium chloride.

**Table 2:** Effects of variation in the concentration of Fe_3_O_4_-NPs on WBDF properties.

Properties	WBDF	1% Fe_3_O_4_ NPs	10% Fe_3_O_4_ NPs	50% Fe_3_O_4_ NPs
PV (mpa.s)	14	18	20	20.5
AV (mpa.s)	19	21	23	24
YP (pa)	15	16	18	19
10s GS (pa)	8	6.3	7.3	8.3
10m GS (pa)	11	9	10.5	11
30 mn filtrate (mL)	9	4.8	6.2	6.8
Filter cake thickness (mm)	2.31	1.81	1.94	2.16
Filter cake thickness (inch)	0.0787	0.0393	0.3937	0.7874

PV: plastic viscosity AV: apparent viscosity YP: yield point GS: gel strength.

## Data Availability

Not Applicable.
